# Association of inflammatory markers with depression and anxiety in female patients with primary Sjögren’s syndrome

**DOI:** 10.11613/BM.2025.030701

**Published:** 2025-08-15

**Authors:** Fanika Mrsić, Ines Vukasović, Andrea Tešija Kuna, Blaženka Ladika Davidović, Jasenka Markeljević

**Affiliations:** 1 Department of Clinical Immunology and Rheumatology, Sestre milosrdnice University Hospital Center, Zagreb, Croatia; 2 Department of Clinical Chemistry, Sestre milosrdnice University Hospital Center, Zagreb, Croatia; 3 School of Medicine, Catholic University of Croatia, Zagreb, Croatia; 4 Department of Oncology and Nuclear Medicine, Sestre milosrdnice University Hospital Center, Zagreb, Croatia; 5 School of Medicine, University of Zagreb, Zagreb, Croatia

**Keywords:** anxiety, depression, inflammation, interleukin 6, primary Sjögren’s syndrome

## Abstract

**Introduction:**

Primary Sjögren’s syndrome (pSS) is a chronic autoimmune disease affecting exocrine glands and is frequently accompanied by depression and anxiety. Proinflammatory cytokines, particularly interleukin 6 (IL-6), have been implicated in the pathogenesis of both pSS and mood disorders. This study aimed to assess the association between inflammatory markers, disease activity, and psychological symptoms in patients with pSS.

**Materials and methods:**

A cross-sectional study was conducted on 60 female patients diagnosed with pSS at Sestre milosrdnice University Hospital Center between 2019 and 2021. Depression and anxiety were evaluated using the Hospital Anxiety and Depression Scale. Inflammatory biomarkers (erythrocyte sedimentation rate, rheumatoid factor, ferritin, fibrinogen, CRP, C3, C4, IL-6) and disease activity indices (ESSDAI, ESSPRI) were analyzed. Statistical analyses, including logistic regression, were applied to determine independent predictors of depression and anxiety.

**Results:**

Depression was detected in 39/60 of patients, while 34/60 exhibited anxiety symptoms. Patients with either depression or anxiety had significantly higher IL-6 concentration (P < 0.001 and P = 0.002, respectively). Logistic regression identified IL-6 as an independent predictor of depression (OR = 3.23, 95%CI: 1.07 - 9.80, P = 0.038), while ESSPRI fatigue was a significant predictor of anxiety (OR = 2.01, 95%CI: 1.13 – 3.58, P = 0.018).

**Conclusions:**

The findings suggest that IL-6 could be a predictor of pSS-related depression, potentially serving as a biomarker for this extraglandular manifestation and ESSPRI fatigue as a predictor for anxiety.

## Graphical abstract

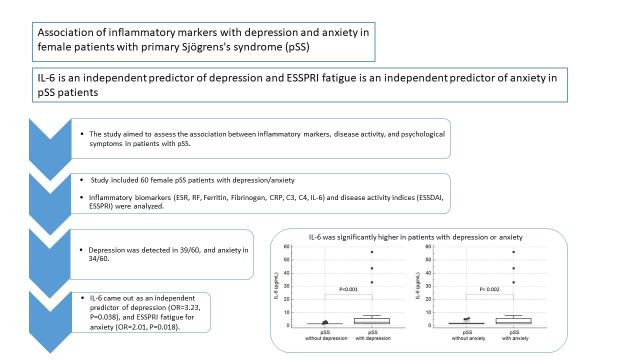

## Highlights

• More than half of patients with primary Sjögren’s syndrome have depression and anxiety

• Interleukin 6 concentration is higher in primary Sjögren’s syndrome patients with either depression or anxiety

• Interleukin 6 may be a predictor of depression in patients with primary Sjögren’s syndrome

• European League Against Rheumatism Sjögren’s Syndrome Patient Reported Index (ESSPRI) for fatigue may be a predictor of anxiety in patients with primary Sjögren’s syndrome

## Introduction

Sjögren’s syndrome (SS) is a chronic systemic, autoimmune, lymphoproliferative disease affecting exocrine glands and internal organs (*1*). The female-to-male ratio of SS is 9:1. It is clinically manifested as primary SS (pSS), with glandular and systemic manifestations, or secondary SS (sSS) associated with other autoimmune diseases (*1*). It is characterized by dryness of the mouth and eyes with the production of various autoantibodies in the blood (*2*). In addition to the symptoms associated with functional impairment of the glands with exocrine secretion and extraglandular symptoms, patients with pSS may also have general symptoms such as depression (*3*). Although the exact etiopathogenetic mechanism of SS is not known, it is believed that disturbed neuroendocrine-humoral regulation of the immune system in genetically predisposed individuals exposed to provocative factors plays a role in the development of pSS (*4*). Despite evidence of a strong genetic component, the interaction of the immune, endocrine and nervous systems, mediated by hormones, neurotransmitters, neuropeptides, cytokines and their receptors, is key in the development of SS (*4*). It is believed that the imbalance of cytokines in pSS causes gland dysfunction and systemic manifestations, which results in decreased secretory function of gland (*5*). In addition to symptoms associated with gland dysfunction, interleukin 6 (IL-6) may play a role in the development of depression (*6*). Its overexpression represents a potential biological pathway by which inflammation may contribute to symptoms of depression. There are several proposed mechanisms by which cytokines could influence the development of depression. Cytokines can activate the hypothalamic-pituitary-adrenal (HPA) axis, directly, *via* corticotropin-releasing hormone (CRH) or *via* cytokine-induced resistance of glucocorticoid receptors (*7*). This causes hyperactivity of the HPA axis due to impaired negative feedback. Furthermore, proinflammatory cytokines can alter monoamine neurotransmitters in different regions of the brain leading to an increase in acute phase proteins and serum concentration of IL-6 (*7*). This leads to a reduced availability of L-tryptophan, which is known to reduce the availability of serotonin in the central nervous system. Interleukin 6 is a proinflammatory cytokine that is activated in response to tissue injury and infection. It contributes to host defense by stimulating acute phase reactants such as C-reactive protein (CRP), ceruloplasmin, haptoglobin and ferritin. Its increased concentration was found in the saliva, tears and serum of patients with SS. Also, it was shown that concentration of IL-6 correlates with dryness of the eyes and mucous membranes, with the degree of lymphocytic infiltration of the salivary glands and with the number of extraglandular symptoms (*8*). Interleukin 6 mediates its biological effects through the IL-6 receptor (IL-6R). Activation of IL-6R leads to the maturation of B cells, which favors their hyperplasia and increased concentration of inflammatory reactants, which explains the proinflammatory state visible in SS (*6*, *9*).

The most recent studies have shown an increased concentration of IL-6 in the blood of patients with depression and a positive correlation between the concentration of cytokines in the cerebrospinal fluid with disease activity in depressed patients (*10*, *11*). Considering the above, a potential therapeutic goal could be to block IL-6 or its receptor in pSS, which would reduce systemic and local inflammation, reduce the activation of B cells and autoantibodies, and thus have a favorable effect on the local inflammatory process and systemic autoimmunity in pSS.

Depression and anxiety are common comorbidities in patients with pSS. We hypothesized that certain inflammatory markers or indicators of disease activity could potentially serve as predictors for developing these psychological symptoms. The aim of this study was to determine the association of inflammatory markers, and activity of disease with depression and anxiety in patients with pSS.

## Materials and methods

### Subjects

This cross-sectional study included 60 female patients diagnosed with pSS. The patients were recruited from the population of patients with pSS treated at the Department of Clinical Immunology and Rheumatology, Sestre milosrdnice University Hospital Center during two years of research (from 2019 to 2021). The diagnosis of pSS was made based on the classification criteria for pSS: American College of Rheumatology (ACR) and the European League Against Rheumatism (EULAR) (*12*). All patients with pSS fulfilled the classification criteria for pSS, which requires the presence of either anti-Ro60/SS-A and/or a positive biopsy finding of the minor labial salivary glands (focus score ≥ 1). An exclusion factor was the association or overlapping of pSS with another autoimmune disease (rheumatoid arthritis, systemic lupus erythematosus, systemic sclerosis, mixed connective tissue disease, primary biliary cirrhosis, chronic active hepatitis, Hashimoto’s thyroiditis), other acute and chronic inflammatory diseases, patients with a psychiatric diagnosis before the diagnosis of SS, patients treated with anti-inflammatory drugs, corticosteroids, antidepressants, antipsychotics, hormone replacement therapy within 6 weeks before inclusion in the trial. Disease activity and subjective complaints of patients were measured using two indices defined and validated in clinical practice; European League Against Rheumatism - EULAR Sjögren’s Syndrome Disease Activity Index (ESSDAI) and EULAR Sjögren’s Syndrome Patient Reported Index (ESSPRI) (*13*, *14*). ESSDAI is a standardized instrument for the homogenous evaluation of systemic activity in order to be used as outcome criteria to evaluate primary SS in clinical trials as well in daily practice. It includes 12 different domains (*i.e.* organ systems: cutaneous, respiratory, renal, articular, muscular, peripheral nervous system (PNS), central nervous system (CNS), hematological, glandular, constitutional, lymphadenopathic, biological). Disease activity is classified as low (ESSDAI < 5), moderate activity (ESSDAI 5 - 13), and high activity (ESSDAI ≥ 14). The ESSPRI scale consists of three individual 0-10 cm scales and includes a domain representing the patient’s symptoms: dryness, pain and fatigue. The total maximum score is 30, with a higher score reflecting more severe complaints. A test that can objectivize dry eye is called the Schirmer test. It measures the 5-minute wetting of standardized filter paper placed in the lower lateral part of the fornix of the eyelid and serves as an indicator of basal (anesthetized) or stimulated (non-anesthetized) tear flow. The speed of movement along the test strip is proportional to the rate of tear secretion. Schirmer test value without anesthesia of 5 mm or less in both eyes is one of the classification criteria for SS. Salivary flow measurement (sialometry) is used for assessment of dry mouth. It is used to measure the amount of saliva produced. Sialometry in SS, as one of the diagnostic criteria, collects unstimulated saliva for 15 minutes and a 5-minute saliva collection after chewing paraffin to ensure salivary stimulation. An unstimulated whole saliva flow rate of less than 0.1 mL/min is considered abnormal and is included in the criteria for SS. As a screening for the presence of clinically significant symptoms of anxiety and depression, patients completed the Hospital Anxiety and Depression Scale (HADS) (*15*). It is a self-report rating scale of 14 items (range 0-3) that consists of 7 items for anxiety and 7 items for depression. The score may range between 0 and 21 per subscale (0-7 absence of depression/anxiety, 8-10 mild, 11-14 moderate and 15-21 severe depression/anxiety). Before the very beginning, the research received the consent of the Ethics Committee of Sestre milosrdnice University Hospital Center (EP-18647/18-5) and the informed consent of all participants. The research was conducted in accordance with the Declaration of Helsinki.

### Methods

For patients with pSS following analysis were performed: complete blood count, erythrocyte sedimentation rate (ESR), C-reactive protein (CRP), rheumatoid factor (RF), antinuclear antibodies (ANA), anti-Ro60/SS-A and anti-La/SS-B antibodies, complement components (C3, C4), ferritin, fibrinogen and IL-6. Analysis was conducted in Department of Clinical Chemistry and Department of Oncology and Nuclear Medicine, Sestre milosrdnice University Hospital Center. Blood samples were collected during morning hours between 7 and 9 in standardized collection tubes: with clot activator for biochemistry, K2EDTA for complete blood count, 3.8% Na-citrate (1:5) for ESR, and 3.2% Na-citrate (1:10) for fibrinogen (all Vaccuette, Greiner Bio-One, Kremsmünster, Austria). Complete blood count was analyzed on the Sysmex XN1000 hematology analyzer (Sysmex Corp., Kobe, Japan), ESR manually with modified Westergren method and fibrinogen assay was performed on the BCS XP System hemostasis analyzer (Siemens Healthineers, Erlangen, Germany). Rheumatoid factor, CRP, C3, C4 and ferritin were measured on the Abbott Architect c8000 analyzer (Abbott Laboratories, Abbott Park, USA). Detection of ANA was performed using indirect immunofluorescence test (IIF) on Human Epithelial Type 2 cells substrate (HEp-2) (Euroimmun, Luebeck, Germany) using Olympus BX43 fluorescence microscope (Olympus, Tokyo, Japan), anti-Ro60/SS-A and anti-La/SS-B autoantibodies by chemiluminescence immunoassay (CMIA) on Bio-FLASH analyzer (Inova Diagnostics, San Diego, USA).

Interleukin 6 was determined by the electrochemiluminescence method (ECLIA) on a Cobas e601 analyzer (Roche Diagnostics, Basel, Switzerland). The specified measurement range is 1.5 to 5000 pg/mL.

### Statistical analysis

In the analysis of the normality of the distribution of continuous data, the Smirnov-Kolmogorov test was used. The data were presented as median and interquartile range (IQR), with minimum and maximum if applicable. According to the obtained results, in the further analysis, appropriate non-parametric tests were used.

Antibody positivity, ESSDAI, and HADS categories were presented as the number of patients out of the total number.

Differences in continuous variables between groups were analyzed by the Mann-Whitney U test. Logistic regression analysis was done and presented with odds ratio (OD), corresponding 95% confidence interval (OR, 95%CI), and P values. All values that in univariate analysis had statistical significance for the OR were entered into a multivariate regression prediction model. Multivariate model was used for the prediction of depression and anxiety and additionally was adjusted for age.

P values less than 0.05 were considered significant. For statistical analysis, MedCalc Statistical Software version 20.008 (MedCalc Software, Ostend, Belgium) was used.

## Results

The study included 60 patients diagnosed with pSS. All participants were female, with an average age of 58 years in the range of 21-75 years. Fifty-nine out of 60 patients had a positive Schirmer test, while 45/60 had an unstimulated salivary flow ≤ 0.1 mL/min. Salivary gland biopsy (≥ 1 focus/4 mm^2^) was positive in 26/60 patients. Descriptive statistics of examined clinical and laboratory values in patients are shown in Table 1.

**Table 1:** Descriptive statistics of examined clinical and laboratory values in pSS patients (N = 60)

		**Min**	**Max**
Age (years)	58 (51-64)	21	75
ESSDAI	4 (2-7)	0	15
Duration of the disease from the onset of symptoms (years)	9 (5-13)	2	21
Duration of disease since diagnosis (years)	7 (3-10)	1	20
ESSPRI total	21 (17-23)	6	28
ESSPRI dryness	7 (6-8)	1	10
ESSPRI pain	7 (5-8)	1	10
ESSPRI fatigue	7,00 (5-8)	1	10
HADS total	17 (12-22)	3	27
HADS depression	9 (6-12)	2	14
HADS anxiety	8 (5-11)	1	14
ESR (mm/3.6 ks)	18 (9-32)	2	71
CRP (mg/L)	1,90 (1,0-5,6)		
RF (IU/mL)	20 (20-73)		
Ferritin (µg/L)	58.0 (19.3-117.0)		
Fibrinogen (g/L)	3.5 (3.0-4.6)		
C3 (g/L)	1.20 (1.07-1.41)		
C4 (g/L)	0.25 (0.20-0.31)		
IL-6 (pg/mL)	1.85 (1.50-3.80)		
**Antibodies positivity**	**N/total**		
ANA positive	52/60		
anti-Ro60/SS-A positive	37/60		
anti-La/SS-B positive	18/60		
**ESSDAI categories**	**N/total**		
Low disease activity	31/60		
Moderate disease activity	25/60		
High disease activity	4/60		
**HADS depression categories**	**N/total**		
absence of depression	21/60		
mild depression	19/60		
moderate depression	20/60		
severe depression	0/60		
**HADS anxiety categories**	**N/total**		
absence of anxiety	26/60		
mild anxiety	19/60		
moderate anxiety	15/60		
severe anxiety	0/60		
Data are presented as median (interquartile range). ESSDAI - EULAR Sjögren´s Syndrome Disease activity. ESSPRI - EULAR Sjögren´s Syndrome Patient Reported Index. HADS - Hospital Anxiety and Depression Scale. ESR - erythrocyte sedimentation rate. CRP - C-reactive protein. RF - rheumatoid factor. C3 - complement component C3. C4 - complement component C4. IL-6 - interleukin-6.			

Twenty patients (20/60) exhibited neither depression, nor anxiety. Frequencies of depression and anxiety categories in pSS patients are presented in Table 1. There was no patient with severe depression and anxiety (HADS score > 14).

Among the 39 patients diagnosed with depression, 33/39 also met criteria for comorbid anxiety. Within the cohort presenting with both depression and anxiety, the majority were classified within the same HADS severity category: 13/33 patients demonstrated mild depression and mild anxiety, and 14/33 patients demonstrated moderate depression and moderate anxiety. Discordant severity levels were observed in 6 patients, with 5 patients exhibiting moderate depression and mild anxiety, and 1 patient exhibiting mild depression and moderate anxiety. Isolated depression was identified in 6 patients, of whom 5 were classified with mild depression and 1 with moderate depression. Only one patient exhibited isolated anxiety, categorized as mild according to HADS criteria.

**Figure 1:**
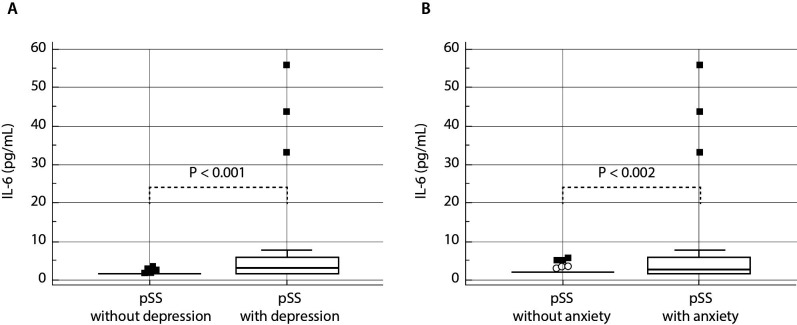
Difference in IL-6 concentration (pg/mL) between pSS patients: a) with (N = 39) and without (N = 21) depression (P < 0.001); b) with (N = 34) and without anxiety (N = 26) (P = 0.002), (Mann Whitney U test, P < 0.05 was considered statistically significant).

The results revealed that pSS patients with either depression or anxiety (regardless of HADS category) had higher concentration of IL-6 (P < 0.001, P = 0.002, respectively) (Figure 1).

Median (IQR) of IL-6 concentration in patients with depression was 2.8 (1.58-5.58) pg/mL in comparison to 1.50 (1.50-1.53) pg/mL in patients without depression, and 2.40 (1.50-5.60) pg/mL in patients with anxiety compared to 1.50 (1.50-2.00) pg/mL in patients without anxiety.

In the univariate analysis, IL-6, CRP, ESSPRI total, ESSPRI pain, and ESSPRI fatigue were statistically significant for the OR for depression. These variables were subsequently included in a multivariate logistic regression model to obtain more accurate and reliable estimates of their association with depression. Only IL-6 was identified as an independent predictor of depression (OR = 3.45, 95%CI: 1.12-10.62, P = 0.031; Table 2).

**Table 2:** Logistic regression analysis for the identification of dependency of depression (according to the Hospital Anxiety and Depression Scale, HADS) on one or more independent variables (marker of inflammation, disease duration from the symptom onset, European League Against Rheumatism - EULAR Sjögren’s Syndrome Disease Activity Index (ESSDAI), EULAR Sjögren’s Syndrome Patient Reported Index (ESSPRI) and age)

	**Depression**					
**Variable**	**Univariate logistic regression analysis**		**Multivariate logistic regression analysis**		**Multivariate logistic regression analysis after adjustment for age**	
	**OR (95% CI)**	**P**	**OR (95%CI)**	**P**	**OR (95%CI)**	**P**
IL-6	3.58 (1.31-9.75)	0.013	3.45 (1.12-10.62)	0.031	3.23 (1.07-9.80)	0.038
ESR	1.02 (0.98-1.06)	0.275				
RF	0.99 (0.98-1.00)	0.085				
Ferritin	1.00 (1.00-1.01)	0.407				
Fibrinogen	1.93 (0.97-3.81)	0.060				
CRP	1.25 (1.01-1.54)	0.037	1.00 (0.80-1.24)	0.975	0.98 (0.78-1.22)	0.840
C3	9.08 (0.73-112.77)	0.086				
C4	23.28 (0.03-20514.03)	0.363				
ESSDAI	0.63 (0.27-1.48)	0.287				
ESSPRI total	1.23 (1.07-1.41)	0.004	1.04 (0.70-1.53)	0.862	1.03 (0.70-1.52)	0.885
ESSPRI pain	1.42 (1.08-1.87)	0.013	1.09 (0.60-1.98)	0.788	1.09 (0.59-2.00)	0.783
ESSPRI fatigue	1.60 (1.18-2.16)	0.002	1.36 (0.77-2.39)	0.293	1.39 (0.79-2.47)	0.255
ESSPRI dryness	1.16 (0.89-1.51)	0.286				
Disease duration from the symptom onset	1.13 (1.00-1.28)	0.058				
Age	1.05 (0.99-1.11)	0.104			1.02 (0.95-1.10)	0.540
OR (95%CI) - odds ratio with corresponding 95% confidence interval. IL-6 - interleukin-6. ESR - erythrocyte sedimentation rate. RF - rheumatoid factor. CRP - C-reactive protein. C3 - complement component C3. C4 - complement component C4. ESSDAI - European League Against Rheumatism - EULAR Sjögren’s Syndrome Disease Activity Index. ESSPRI - EULAR Sjögren´s Syndrome Patient Reported Index. P < 0.05 was considered statistically significant.						

After adjusting for age, IL-6 remained a significant independent predictor (OR = 3.23, 95%CI: 1.07-9.80, P = 0.038; Table 2). The age-adjusted multivariate model achieved an area under the curve (AUC) of 0.88 (95%CI: 0.77-0.95) and correctly identified 80% of patients with depression. Adjustment for age was performed to exclude possible bias due to the modifying influence of age on the relationship between variables.

The same set of tests was applied for anxiety. In the multivariate logistic regression analysis, which included IL-6, CRP, ESSPRI total, ESSPRI pain, ESSPRI fatigue, and ESSDAI, only ESSPRI fatigue was identified as an independent predictor of anxiety (OR = 1.99, 95%CI: 1.12-3.53, P = 0.019; Table 3). This association remained significant after adjustment for age (OR = 2.01, 95%CI: 1.13-3.58, P = 0.018; Table 3).

**Table 3:** Logistic regression analysis for the identification of dependency of anxiety (according to the Hospital Anxiety and Depression Scale, HADS) on one or more independent variables (marker of inflammation, disease duration from the symptom onset, European League Against Rheumatism - EULAR Sjögren’s Syndrome Disease Activity Index (ESSDAI), EULAR Sjögren’s Syndrome Patient Reported Index (ESSPRI) and age)

	**Anxiety**					
**Variable**	**Univariate logistic regression analysis**		**Multivariate logistic regression analysis**		**Multivariate logistic regression analysis after adjustment for age**	
	**OR (95%CI)**	**P**	**OR (95%CI)**	**P**	**OR (95%CI)**	**P**
IL-6	3.58 (1.31-9.75)	0.013	1.38 (0.91-2.08)	0.130	1.32 (0.86-2.01)	0.205
ESR	1.02 (0.98-1.06)	0.273			0.97 (0.69-1.35)	
RF	1.00 (0.99-1.00)	0.367				
Ferritin	1.00 (1.00-1.01)	0.681				
Fibrinogen	1.60 (0.86-2.96)	0.137				
CRP	1.08 (0.97-1.20)	0.160				
C3	2.69 (0.29-24.80)	0.382				
C4	1.26 (0.00-716.53)	0.944				
ESSDAI	0.97 (0.85-1.11)	0.662				
ESSPRI total	1.23 (1.07-1.41)	0.003	0.97 (0.70-1.34)	0.830	0.97 (0.69-1.35)	0.836
ESSPRI pain	1.31 (1.01-1.69)	0.043	0.98 (0.60-1.60)	0.932	0.97 (0.59-1.60)	0.896
ESSPRI fatigue	1.90 (1.33-2.73)	0.001	1.99 (1.12-3.53)	0.019	2.01 (1.13-3.58)	0.018
ESSPRI dryness	1.13 (0.87-1.46)	0.367				
Disease duration from the symptom onset	1.08 (0.96-1.20)	0.191				
Age	1.03 (0.98-1.09)	0.263			1.02 (0.96-1.09)	0.511
OR (95%CI) - odds ratio with corresponding 95% confidence interval. IL-6 - interleukin-6. ESR - erythrocyte sedimentation rate. RF - rheumatoid factor. CRP - C-reactive protein. C3 - complement component C3. C4 - complement component C4. ESSDAI - European League Against Rheumatism - EULAR Sjögren’s Syndrome Disease Activity Index. ESSPRI - EULAR Sjögren´s Syndrome Patient Reported Index. P < 0.05 was considered statistically significant.						

The resulting age-adjusted model yielded an AUC of 0.85 (95%CI: 0.73-0.93) and correctly identified 75% of patients with anxiety.

## Discussion

Our results confirmed that depression and anxiety are accompanied in more than half of patients within our group of pSS patients. Among all tested parameters only IL-6 came out as an independent predictor for depression while ESSPRI fatigue was an independent predictor for anxiety in pSS.

Interleukin 6 as a proinflammatory cytokine plays an important role in the immune response of inflammatory diseases (*6*, *16*, *17*). Considering the results of previous studies showing increased concentration of IL-6 in some other inflammatory rheumatic diseases such as systemic lupus erythematosus, rheumatoid arthritis and systemic sclerosis, it is assumed that IL-6 could be an indicator of symptoms common to all inflammatory rheumatic diseases such as depression (*18*, *19*). Although the exact mechanism of pathogenesis is not known, a large number of studies have observed a connection between concentration of IL-6 and depression (*20*). The fact that IL-6 represents a probable biological pathway through which inflammation can contribute to symptoms of depression becomes important in clinical practice in patients with pSS (*20*, *21*). A small number of studies investigated the association between depression and inflammatory markers in patients with pSS (*22*). The results of our study showed that among investigated markers, IL-6 concentration could be used as an independent predictor of depression in patients with pSS even after adjusting for age since some studies have shown that IL-6 concentration increases with age, although this was not proven in others (*23*, *24*). Our result is consistent with data obtained from earlier studies, noting that musculoskeletal pain and/or some other, yet unexplored, mechanism may cause psychological disturbances such as fatigue and depression in patients with pSS (*20*, *25*). It is still not clear whether these non-specific symptoms are the cause or consequence of a chronic inflammatory disease. In addition to genetic predisposition, exposure to life stress, which is a prominent risk factor for depression, induces an inflammatory response and elevation of inflammatory markers including proinflammatory cytokines (*26*).

Although some studies have shown increased concentration of IL-6 in pSS, very few studies have investigated its association with disease activity measured by ESSDAI and ESSPRI (*21*, *27*, *28*). Our results did not reveal the predictive value of disease activity indices ESSPRI and ESSDAI for depression, while ESSPRI fatigue could be a predictor for anxiety. Some studies have shown that fatigue, depression and anxiety are more pronounced in patients with higher disease activity, but the same has not been proven in other studies (*29*-*31*). This disparity and heterogeneity of results indicates that the systemic manifestations of the disease and the subjective symptoms are two different components of the disease and should be assessed separately as such. Based on our results we can conclude that depression is not influenced by objective clinical indicators, which speaks in favor of a complex, still unexplained pathophysiological and biopsychosocial mechanisms of depression and anxiety in patients with pSS.

This study has several limitations. It is a single-center research that included a small number of patients. Regarding the incidence difference of pSS for females and males there are no male participants intentionally. We tried to minimize possible gender differences by using the female population in the research. However, this may cause that main findings may not apply to the men population with pSS. According to our obtained data, it can be assumed that IL-6 can influence the onset of depression in patients with pSS, with the note of potential bidirectional relationship of inflammation and depression and that patients with pSS may also have a genetic basis for the onset of depression. Our research is cross-sectional study and, unlike longitudinal research, does not enable within a patient’s comparison, knowing that cytokine concentration can vary significantly within a short period of time. Although cytokines can influence the onset and consequent disease activity, the disease activity itself can affect the concentration of cytokines and other inflammatory markers as part of a two-way negative feedback loop.

In conclusion, our results support a potential proinflammatory etiopathogenetic model for the risk of developing depressive symptoms in patients with pSS suggesting that IL-6 could be a predictor of pSS-related depression. Among clinical activity indices, ESSPRI fatigue could be recognized as a predictor for pSS-related anxiety. We believe that these results will contribute to a better understanding of the occurrence of this frequent and limiting comorbidity, thereby facilitating the diagnosis and treatment of depression in patients with pSS.

## Data Availability

The data generated and analyzed in the presented study are available from the corresponding author on request.
